# Social Disparities in Outpatient and Inpatient Management of Pediatric Supracondylar Humerus Fractures

**DOI:** 10.3390/jcm11154573

**Published:** 2022-08-05

**Authors:** Jacob M. Modest, Peter G. Brodeur, Kang W. Kim, Edward J. Testa, Joseph A. Gil, Aristides I. Cruz

**Affiliations:** Department of Orthopaedic Surgery, Alpert Medical School of Brown University, Providence, RI 02903, USA

**Keywords:** supracondylar humerus fracture, SCHF, pediatric, disparity, social deprivation

## Abstract

Socioeconomic status, race, and insurance status are known factors affecting adult orthopaedic surgery care, but little is known about the influence of socioeconomic factors on pediatric orthopaedic care. The purpose of this study was to determine if demographic and socioeconomic related factors were associated with surgical management of pediatric supracondylar humerus fractures (SCHFs) in the inpatient versus outpatient setting. Pediatric patients (<13 years) who underwent surgery for SCHFs were identified in the New York Statewide Planning and Research Cooperative System database from 2009–2017. Inpatient and outpatient claims were identified by International Classification of Diseases-9-Clinical Modification (CM) and ICD-10-CM SCHF diagnosis codes. Claims were then filtered by ICD-9-CM, ICD-10-Procedural Classification System, or Current Procedural Terminology codes to isolate SCHF patients who underwent surgical intervention. Multivariable logistic regression analysis was performed to determine the effect of patient factors on the likelihood of having inpatient management versus outpatient management. A total of 7079 patients were included in the analysis with 4595 (64.9%) receiving inpatient treatment and 2484 (35.1%) receiving outpatient treatment. The logistic regression showed Hispanic (OR: 2.386, *p* < 0.0001), Asian (OR: 2.159, *p* < 0.0001) and African American (OR: 2.095, *p* < 0.0001) patients to have increased odds of inpatient treatment relative to White patients. Injury diagnosis on a weekend had increased odds of inpatient management (OR: 1.863, *p* = 0.0002). Higher social deprivation was also associated with increased odds of inpatient treatment (OR: 1.004, *p* < 0.0001). There are disparities among race and socioeconomic status in the surgical setting of SCHF management. Physicians and facilities should be aware of these disparities to optimize patient experience and to allow for equal access to care.

## 1. Introduction

In an effort to reduce healthcare costs and resource consumption, and thereby increase healthcare value, there has been a recent transition of surgical management from the inpatient to the outpatient setting across multiple fields [1,2]. For example, outpatient care of pediatric orthopaedic day surgeries produced a cost savings of up to 43%, which was attributed to reduced operating time and improved resource utilization [3]. In evaluating the consequent benefits of this transition towards outpatient surgical care, pediatric supracondylar humerus fractures (SCHF) are a specific area of interest both due to their high prevalence and their frequent need for surgical intervention [4]. While operative Gartland Type III SCHFs are oftentimes admitted for urgent surgical management, prior studies have documented that Gartland Type II fractures can be appropriately treated in an outpatient setting [5,6]. Multiple studies have found no difference in complication rates depending on the timing of the treatment, thus suggesting the safety of outpatient management of Type II SCHFs [7,8,9].

Prior orthopaedic literature explored disparities in surgical care for adult patients receiving total knee arthroplasty and anterior cruciate ligament surgery, among others [10,11]. Such studies emphasize disparities in access to care due to factors such as race and ethnicity, often with non-White and Hispanic patients facing the greatest barriers or worse outcomes. Other studies on pain management and orthopaedic care have similarly shown that patients of lower socioeconomic status and those on government-sponsored health insurance also face significant barriers to care [12,13]. Given the disparities of access to care shown in other adult surgeries, it is possible that similar disparities exist in the care of pediatric patients, limiting access to outpatient treatment despite the potential benefits and improved patient satisfaction that outpatient care can provide [14]. Because the association between patient factors and the likelihood of receiving inpatient or outpatient care is comparatively unexplored, we sought to investigate the relationship between pediatric patient demographics and characteristics, and the likelihood of receiving inpatient treatment for pediatric SCHFs.

## 2. Materials and Methods

The New York Statewide Planning and Research Cooperative System (SPARCS) database was used to identify pediatric patients <13 years old who underwent surgery for SCHFs between the years of 2009–2017. SPARCS is an insurance database which includes both inpatient and hospital-associated outpatient (emergency department, ambulatory surgery, and hospital-based clinic visits) claims in New York State. These claims are filed by the patient’s International Classification of Diseases (ICD) diagnosis codes and ICD/Current Procedural Terminology (CPT) procedure codes. IRB approval was not required for this study given the use of a de-identified, publicly available database.

Inpatient and outpatient claims were analyzed by ICD-9 Clinical Modification (CM) and ICD-10 CM SCHF diagnosis codes. These claims were filtered by ICD-9-CM, ICD-10 Procedural Classification System, or CPT codes to identify SCHFs that underwent surgical intervention (refer to Supplemental Tables S1 and S2 for codes utilized). The first operation for any patient was included for analysis.

### Statistical Analyses

Patients were divided into inpatient and outpatient cohorts. Patient demographics were compared amongst cohorts using chi-squared analysis. Mann–Whitney U tests were used when continuous data were found to be not normally distributed. A multivariable logistic regression was performed to assess the likelihood and odds ratio of receiving inpatient treatment using the variables: patient age, sex, race/ethnicity, Social Deprivation Index (SDI), day of diagnosis for surgery, and primary insurance type. “Other race” is defined as other races/ethnicities excluding White, Hispanic, Asian, and African American, but includes multiracial patients. SDI as described by Butler et al. was linked to each patient based on ZIP code. SDI provides a quantitative measure of social determinants of health by converting the following categories to an index from 1–100: percent living in poverty, percent with less than 12 years of education, percent single parent household, percent living in rented housing unit, percent living in overcrowded housing unit, percent of households without a car, and percent non-employed adults under 65 years of age. A higher SDI score equates to increased social deprivation. SDI data in this study were based on 2015 statistics [15]. A *p*-value < 0.05 was considered significant across all statistical analyses. All analyses were performed using SAS 9.4 (SAS Inc., Cary, NC, USA).

## 3. Results

In total, 7079 pediatric patients with SCHFs were included in the analysis with 4595 (64.9%) receiving inpatient treatment and 2484 (35.1%) receiving outpatient treatment. The range for the number of inpatient cases captured per year was 390–647 and 158–374 for outpatient. There was a significant trend towards outpatient treatment over the study period (*p* < 0.0001). 

Multiple demographic differences were noted amongst the groups that underwent inpatient versus outpatient treatment. The inpatient cohort included patients with younger ages and higher social deprivation (Table 1). Inpatient management also had increased likelihood of male sex, Hispanic ethnicity, Asian and African American races, and a weekend injury diagnosis. On the contrary, the outpatient group had increased likelihood of female sex, White race, and weekday injury diagnosis (Table 1).

**Figure 1:**
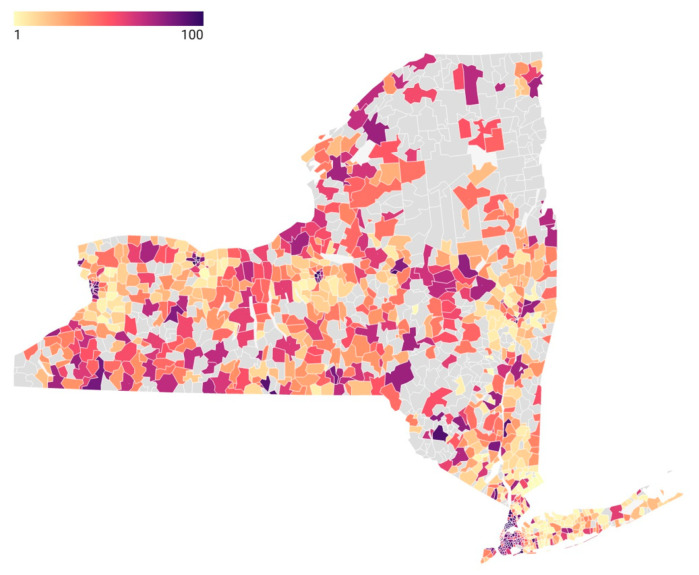
SDI scores by ZIP code in New York (1 to 100). Higher SDI scores represent higher social deprivation. ZIP codes with gray color had no SCHF procedures included in the study.

**Figure 2:**
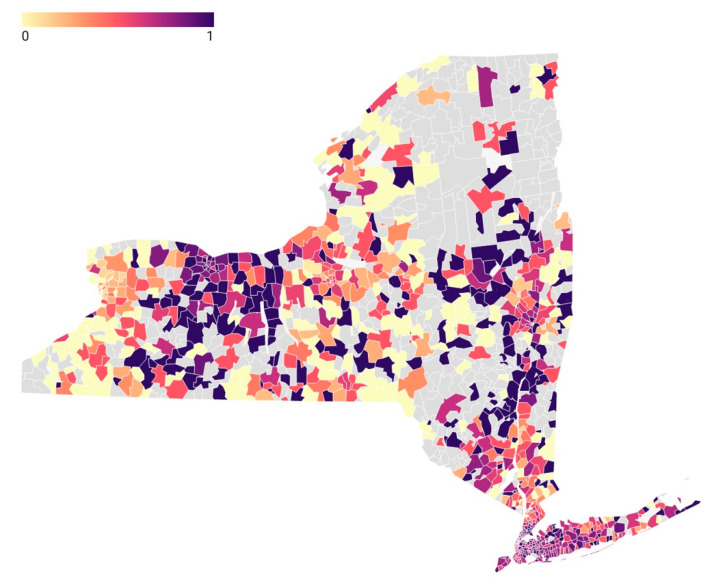
The rate (0–1) of inpatient treatment by ZIP code. A value of 1 represents all procedures were inpatient and a value of 0 represents all procedures were outpatient. ZIP codes with gray color had no SCHF procedures included in the study.

Figure 1 depicts the SDI variation across New York for the ZIP codes with darker areas representing higher social deprivation. There was substantial variation in the SDI throughout the state, with urban areas generally having higher SDI (Figure 1). Figure 2 depicts the percentage of outpatient treatment by ZIP code. As an example, western Long Island, which encompasses New York City and surrounding boroughs, represents higher SDI scores in Figure 1 and higher rates of inpatient treatment in Figure 2. 

The multivariable logistic regression showed Hispanic ethnicity, Asian, African American, and Other races had increased likelihood of inpatient treatment relative to White race. White patients had a 59.2% inpatient treatment rate while Other races had rates above 70%. The group with the highest rate of inpatient management was Hispanic patients at 78.3% (Table 2).

Injury diagnosis on a weekend had increased odds of inpatient management. Federal insurance had decreased odds of inpatient treatment relative to private insurance (Table 2). Lastly, a higher SDI was also associated with increased likelihood of inpatient treatment (Table 2).

## 4. Discussion

The overall trend in pediatric SCHF treatment shows a significant shift towards outpatient care in recent years [17]. This transition parallels other traditionally inpatient orthopaedic procedures that have experienced similar changes including spine, large joint arthroplasty, and trauma with equivalent if not improved clinical results alongside a reduction in cost [14,18,19,20]. Studies on outpatient pediatric orthopaedic procedures, such as those on pediatric anterior cruciate ligament reconstructions, have shown similar benefits in outpatient treatment [21,22]. Furthermore, a 2018 study on pediatric SCHFs have shown no difference in complications or clinical outcomes in outpatient care compared to inpatient care, with significantly reduced operative times and cost in outpatients [23]. Considering the other additional advantages of outpatient treatment, such as fewer scheduling delays and increased autonomy for physicians, the overall transition towards outpatient care for SCHFs is unsurprising [1]. 

It is critical to note that, while outpatient care is becoming more prominent and has been shown to provide quality care with reduced costs for SCHF, it has not been a universal movement for all patients. Importantly, some SCHF may warrant inpatient level of care, such as type III fractures, those with neurovascular compromise, open fracture, or those with concomitant forearm fractures putting the patient as high risk for compartment syndrome. Despite the benefits of outpatient care for the remainder of SCHFs, there remain socioeconomic and demographic factors that affect patient access to healthcare. Our study showed that pediatric patients who were Hispanic, non-White or had increased social deprivation were more likely to receive inpatient care, while those who were White were more likely to receive outpatient treatment for SCHFs. Such results may be potentially due to factors such as language barriers, resource disparity, or geographical issues. While there are no previous studies on the socioeconomic patient characteristic differences between inpatient or outpatient care for pediatric SCHF patients, recent studies on total hip and total knee arthroplasties have emphasized that higher SDI scores are associated with inpatient care, alongside African American race, and existence of comorbidities [16,24]. More specifically, a recent study of adult ambulatory surgical care in New York also reported that Black and Hispanic patients had higher odds of receiving inpatient care [25]. Thus, although this is the first study to explore such disparities in the pediatric orthopaedic population, these trends are likely not unique to pediatric SCHFs and provide insight into the impacts of disparate care with respect to surgical setting. 

There are likely more factors and considerations in evaluating this disparity in surgical setting for SCHF surgical care. Parents and families of the patient may be limited due to constraints of time and location, even if surgeons may support outpatient management of pediatric SCHF. This possibility is supported by a 2015 review of eligibility for inpatient and outpatient care, which reported that a natural requirement for outpatient surgery was a caregiver at home, no history of serious medical problems, and close proximity to the surgical center [26]. It is also important to consider the respective healthcare options of the patients who receive treatment at outpatient centers. While our study found that patients with federal healthcare insurance were more likely to get outpatient care compared to those with private insurance, the currently published literature has shown the opposite. For example, a 2016 study of pediatric SCHF reported that privately insured patients had 2.46 times higher odds of undergoing outpatient surgery [26]. Other studies on pediatric care have shown that Medicaid reimbursements are significantly lower and children insured with Medicaid often have limited access to orthopaedic care nationwide [27]. In contrast, our study showed that federal insurance had an increased chance of receiving outpatient treatment. However, given that the difference in rate of outpatient treatment between federal and private insurance was around 1%, this result, though statistically significant, may not be of clinical significance. 

There are several limitations to this study. As a database study our results rely on the accuracy of medical coding. In addition, we were not able to control for more detailed demographic factors that can be considered to be social determinants of health but were not included in the database, such as occupational demands and cultural or language barriers. We could not account for injury specific factors such as fracture type, fracture severity, or patient pain level which would be expected to influence admission criteria. Prior research shows that delayed care for Gartland Type III fractures may result in higher incidence of compartment syndrome [28] and an increased need for open reduction [28,29]. This may influence the treatment setting decision for Gartland Type III fractures. Therefore, our results may have greater implications in Gartland Type II fractures which have been noted to have minimal differences in complication rates between inpatient and outpatient management [17,23]. Given that we were unable to control for fracture type/severity of injury, an association could theoretically exist amongst a race or ethnicity and fracture severity, and place this group at higher rate of inpatient or outpatient surgery accordingly. Our study only included patients who are residents of New York State, thus national or global trends cannot be directly inferred. However, New York has a very heterogeneous population with high demographic variability among patients, providers, and facilities which improves to generalizability to the United States but limits its generalizability to countries with single-payer or more universal social health systems [30].

This study found that SCHF inpatient management was associated with disparities among race and social deprivation and such findings are likely multifactorial, especially when considering the need for postoperative monitoring of the patient, the availability of a guardian to take care of discharged pediatric patients, the location of the outpatient centers, and healthcare coverage. There are patient and physician benefits to outpatient surgical care that may not be accessible to all patients and it is important to highlight that such disparities exist. Further research needs to examine what other specific factors may be playing a role in inpatient versus outpatient treatment such as fracture pattern, proximity to hospital or surgery center, ease of transportation or public transportation, among others. It is imperative that physicians and facilities be aware of these disparities, discuss them openly, and strive to improve equal access to care and to optimize patient experience and outcome. 

## Figures and Tables

**Table 1:** Patient demographics and characteristics, by primary surgery setting [16].

	Outpatient n = 2484 (35.1%)	Inpatient n = 4595 (64.9%)	*p*-Value
Age, median (mean, SD)	5 (5.4, 2.3)	5 (5.3, 2.4)	**0.033**
SDI, median (mean, SD)	48, (49.4, 31.2)	69 (58.9, 32.8)	**<0.0001**
Race/Ethnicity, n (%)			
White	1648 (66.3)	2175 (47.3)	**<0.0001**
Hispanic	258 (10.4)	930 (20.2)	**<0.0001**
Asian	125 (5)	385 (8.4)	**<0.0001**
African American	167 (6.7)	524 (11.4)	**<0.0001**
Other	286 (11.5)	581 (12.6)	0.1662
Sex, n (%)			
Female	1246 (50.2)	2137 (46.5)	**0.0033**
Male	1238 (49.8)	2458 (53.5)	-
Primary Insurance, n (%)			
Private	2066 (83.2)	3862 (84.1)	0.3407
Federal	345 (13.9)	611 (13.3)	0.4869
Self-pay	73 (2.9)	122 (2.7)	0.4863
Day of Diagnosis			
Weekday	2009 (80.9)	3190 (69.4)	**<0.0001**
Weekend	475 (19.1)	1405 (30.6)	-

**Table 2:** Multivariable logistic regression for the odds of receiving inpatient management for SCHF [16].

	Rate of Inpatient Management (64.9%)	Odds Ratio (95% CI)	*p*-Value
Age	-	1.011 (0.99–1.033)	0.316
Sex			
Males	63.2	-	-
Females *	66.5	0.927 (0.838–1.026)	0.144
Race			
White	59.2	-	-
Hispanic ^φ^	78.3	2.386 (2.022–2.815)	**<0.0001**
Asian ^φ^	75.5	2.159 (1.738–2.681)	**<0.0001**
African American ^φ^	75.9	2.095 (1.716–2.557)	**<0.0001**
Other ^φ^	72.1	1.421 (1.206–1.675)	**<0.0001**
Primary Insurance			
Private	65.2	-	-
Federal ^δ^	63.9	0.831 (0.717–0.964)	**0.0148**
Self-pay ^δ^	62.6	0.853 (0.629–1.157)	0.3054
Day of Diagnosis			
Weekday	61.4		
Weekend ^µ^	74.7	1.863 (1.651–2.101)	**<0.0001**
SDI	-	1.004 (1.002–1.006)	**<0.0001**

* compared to males. ^φ^ compared to White race. ^δ^ compared to private insurance. ^µ^ compared to weekday admission.

## Data Availability

Publically available in the New York Statewide Planning and Research Cooperative System (SPARCS Database).

## Supplementary Materials

The following supporting information can be downloaded at: https://www.mdpi.com/article/10.3390/jcm11154573/s1; Table S1: Diagnosis Codes For Supracondylar Humerus Fractures; Table S2: Procedure Codes For Supracondylar Humerus Fractures.

## References

[1] Crawford D.C., Li C.S., Sprague S., Bhandari M. (2015). Clinical and cost implications of inpatient versus outpatient orthopedic surgeries: A systematic review of the published literature. Orthop. Rev..

[2] Koenig L., Gu Q. (2013). Growth of ambulatory surgical centers, surgery volume, and savings to medicare. Am. J. Gastroenterol..

[3] Fabricant P.D., Seeley M.A., Rozell J.C., Fieldston E., Flynn J.M., Wells L.M., Ganley T.J. (2016). Cost Savings From Utilization of an Ambulatory Surgery Center for Orthopaedic Day Surgery. J. Am. Acad. Orthop. Surg..

[4] Lins R.E., Simovitch R.W., Waters P.M. (1999). Pediatric elbow trauma. Orthop. Clin. N. Am..

[5] Mulpuri K., Wilkins K. (2012). The treatment of displaced supracondylar humerus fractures: Evidence-based guideline. J. Pediatr. Orthop..

[6] Kim W.Y., Chandru R., Bonshahi A., Paton R.W. (2003). Displaced supracondylar humeral fractures in children: Results of a national survey of paediatric orthopaedic consultants. Injury.

[7] Larson A.N., Garg S., Weller A., Fletcher N.D., Schiller J.R., Kwon M., Browne R., Copley L.A., Ho C. (2014). Operative Treatment of Type II Supracondylar Humerus Fractures. Does time to surgery affect complications?. J. Pediatr. Orthop..

[8] Silva M., Wong T.C., Bernthal N.M. (2011). Outcomes of Reduction More Than 7 Days After Injury in Supracondylar Humeral Fractures in Children. J. Pediatr. Orthop..

[9] Bales J.G., Spencer H.T., Wong M.A., Fong Y.-J., Zionts L.E., Silva M. (2010). The Effects of Surgical Delay on the Outcome of Pediatric Supracondylar Humeral Fractures. J. Pediatr. Orthop..

[10] Suarez-Almazor M.E., Souchek J., Kelly P.A., O’Malley K., Byrne M., Richardson M., Pak C. (2005). Ethnic Variation in Knee Replacement. Patient preferences or uninformed disparity?. Arch. Intern. Med..

[11] Testa E.J., Modest J.M., Brodeur P., Lemme N.J., Gil J.A., Cruz A.I. (2022). Do Patient Demographic and Socioeconomic Factors Influence Surgical Treatment Rates After ACL Injury?. J. Racial Ethn. Health Disparities.

[12] Meints S.M., Cortes A., Morais C., Edwards R.R. (2019). Racial and ethnic differences in the experience and treatment of noncancer pain. Pain Manag..

[13] Nayar S.K., Marrache M., Ali I., Bressner J., Raad M., Shafiq B., Srikumaran U. (2020). Racial Disparity in Time to Surgery and Complications for Hip Fracture Patients. Clin. Orthop. Surg..

[14] Rosinsky P.J., Chen S.L., Yelton M.J., Lall A.C., Maldonado D.R., Shapira J., Meghpara M.B., Domb B.G. (2020). Outpatient vs. inpatient hip arthroplasty: A matched case-control study on a 90-day complication rate and 2-year patient-reported outcomes. J. Orthop. Surg. Res..

[15] Butler D.C., Petterson S., Phillips R.L., Bazemore A.W. (2012). Measures of Social Deprivation That Predict Health Care Access and Need within a Rational Area of Primary Care Service Delivery. Health Serv. Res..

[16] Rahman R., Canner J.K., Haut E.R., Humbyrd C.J. (2020). Is Geographic Socioeconomic Disadvantage Associated with the Rate of THA in Medicare-aged Patients?. Clin. Orthop. Relat. Res..

[17] Modest Jacob M., Brodeur Peter Testa Edward J., Lemme Nicholas J., Gil Joseph A., Cruz Artistides I. (2022). Outpatient Operative Management of Pediatric Supracondylar Humerus Fractures: An Analysis of Frequency, Complications, and Cost from 2009 to 2018. J. Pediatr. Orthop..

[18] Adamson T., Godil S.S., Mehrlich M., Mendenhall S., Asher A.L., McGirt M.J. (2016). Anterior cervical discectomy and fusion in the outpatient ambulatory surgery setting compared with the inpatient hospital setting: Analysis of 1000 consecutive cases. J. Neurosurg. Spine.

[19] Makarewich C.A., Stotts A.K., Yoo M., Nelson R.E., Rothberg D.L. (2019). Inpatient Versus Outpatient Treatment of Gartland Type II Supracondylar Humerus Fractures: A Cost and Safety Comparison. J. Pediatr. Orthop..

[20] Qin C., Dekker R.G., Blough J.T., Kadakia A.R. (2016). Safety and Outcomes of Inpatient Compared with Outpatient Surgical Procedures for Ankle Fractures. J. Bone Jt. Surg..

[21] Kadhim M., Gans I., Baldwin K., Flynn J., Ganley T. (2016). Do Surgical Times and Efficiency Differ Between Inpatient and Ambulatory Surgery Centers That are Both Hospital Owned?. J. Pediatr. Orthop..

[22] Patrick N.C., Kowalski C.A., Hennrikus W.L. (2017). Surgical Efficiency of Anterior Cruciate Ligament Reconstruction in Outpatient Surgical Center Versus Hospital Operating Room. Orthopedics.

[23] Rider C.M., Hong V.Y., Westbrooks T.J., Wang J., Sheffer B.W., Kelly D.M., Spence D.D., Flynn J.M., Sawyer J.R. (2018). Surgical Treatment of Supracondylar Humeral Fractures in a Freestanding Ambulatory Surgery Center is as Safe as and Faster and More Cost-Effective Than in a Children’s Hospital. J. Pediatr. Orthop..

[24] Wu M., Belay E., Cochrane N., O’Donnell J., Seyler T. (2021). Comorbidity Burden Contributing to Racial Disparities in Outpatient Versus Inpatient Total Knee Arthroplasty. J. Am. Acad. Orthop. Surg..

[25] Janeway M.G., Sanchez S.E., Chen Q., Nofal M.R., Wang N., Rosen A., Dechert T.A. (2020). Association of Race, Health Insurance Status, and Household Income with Location and Outcomes of Ambulatory Surgery Among Adult Patients in 2 US States. JAMA Surg..

[26] Fletcher N.D., Sirmon B.J., Mansour A.S., Carpenter W.E., Ward L.A. (2016). Impact of insurance status on ability to return for outpatient management of pediatric supracondylar humerus fractures. J. Child. Orthop..

[27] Skaggs D.L., Lehmann C.L., Rice C., Killelea B.K., Bauer R.M., Kay R.M., Vitale M.G. (2006). Access to Orthopaedic Care for Children with Medicaid Versus Private Insurance. Results of a national survey. J. Pediatr. Orthop..

[28] Ramachandran M., Skaggs D.L., Crawford H.A., Eastwood D.M., LaLonde F.D., Vitale M.G., Do T.T., Kay R.M. (2008). Delaying treatment of supracondylar fractures in children: Has the pendulum swung too far?. J. Bone Jt. Surg..

[29] Loizou C.L., Simillis C., Hutchinson J.R. (2009). A systematic review of early versus delayed treatment for type III supracondylar humeral fractures in children. Injury.

[30] Vital Statistics of New York State. https://www.health.ny.gov/statistics/vital_statistics/vs_reports_tables_list.htm.

