# Supplementary material for: Social Disparities in Outpatient and Inpatient Management of Pediatric Supracondylar Humerus Fractures
Source: J Clin Med. 2022 Aug 5;11(15):4573. doi: 10.3390/jcm11154573 (PMC9369519; doi:10.3390/jcm11154573)
Supplement: Supplementary file 1 [file jcm-11-04573-s001.zip › jcm-1823489-supplementary.pdf]

**Supplemental Table S1**. Diagnosis Codes For Supracondylar Humerus Fractures.

| ICD 9 CM Description | ICD 9 CM | ICD 10 CM Equivalent |
|---|---|---|
| Closed supracondylar fracture of humerus | 812.41 | S42.411A, S42.412A, S42.413A, S42.414A, S42.415A, S42.416A, S42.421A, S42.422A, S42.423A, S42.424A, S42.425A, S42.426A |
| Open supracondylar fracture of humerus | 812.51 | S42.411B, S42.412B, S42413B, S42.414B, S42.415B, S42.416B, S42.421B, S42.422B, S42423B, S42.424B, S42.425B, S42.426B |

**Supplemental Table S2**. Procedure Codes For Supracondylar Humerus Fractures.

| | ICD 9 CM | ICD 10 PCS | CPT |
|---|---|---|---|
| Internal Fixation | 78.52, 79.11, 79.31 | 0PHF04Z, 0PHF34Z, 0PHF44Z, 0PHG04Z, 0PHG34Z, 0PHG44Z, 0PHF06Z, 0PHF36Z, 0PHF46Z, 0PHG06Z, 0PHG36Z, 0PHG46Z, 0PSC34Z, 0PSC44Z, 0PSD34Z, 0PSD44Z, 0PSF34Z, 0PSF44Z, 0PSG34Z, 0PSG44Z, 0PSC04Z, 0PSD04Z, 0PSF04Z, 0PSG04Z | 24538, 24545, 24546 |
